# Ultrasound-Guided Coronary Angiography Through a True Superficial Radial Artery in a Patient With an Absent Conventional Radial Pulse: A Case Report

**DOI:** 10.7759/cureus.115903

**Published:** 2026-09-07

**Authors:** Ahmed Abdelkader, Essam Gaber Nosir

**Affiliations:** 1 Cardiology, National Health Service (NHS), Southampton, GBR; 2 Cardiology, National Heart Institute, Cairo, EGY

**Keywords:** anatomical variation, coronary angiography, cto: chronic total occlusion, radial artery anomaly, superficial radial artery, transradial access, ultrasound-guided vascular access

## Abstract

Transradial access (TRA) is the preferred vascular approach for diagnostic coronary angiography and percutaneous coronary intervention, offering lower bleeding rates, fewer vascular complications, earlier ambulation, and greater patient comfort compared with transfemoral access. However, anatomical variations of the radial artery can complicate conventional cannulation and may necessitate alternative access strategies. The true superficial radial artery (SRA) is an uncommon congenital variant in which the radial artery courses superficially over the dorsolateral wrist rather than along its usual deep path, often resulting in an absent or weak pulse at the conventional radial access site. A 62-year-old man with type 2 diabetes mellitus, hypertension, and active smoking, who presented with Canadian Cardiovascular Society (CCS) class II exertional angina. Examination before coronary angiography revealed complete absence of a palpable radial pulse at the conventional volar wrist site, but a distinct pulsation was identified over the dorsolateral aspect of the right wrist. Vascular ultrasound confirmed a true SRA and guided vascular access, allowing successful first-attempt insertion of a 6-French hydrophilic radial sheath. Diagnostic coronary angiography was completed without arterial spasm, bleeding, hematoma, or crossover to femoral access, with a procedure duration of 15 minutes, fluoroscopy time of 10 minutes, contrast volume of 45 mL, and radiation dose of 30 Gy·cm². Coronary angiography demonstrated chronic total occlusion of the left circumflex artery extending into the second obtuse marginal branch, with well-developed collateral circulation from the right coronary artery. The patient was subsequently referred for coronary artery bypass grafting. This case illustrates that careful vascular examination combined with vascular ultrasound can identify a true SRA in patients with an absent conventional radial pulse. Recognizing this rare anatomical variant can help preserve the transradial approach, avoid unnecessary femoral crossover, and allow safe completion of coronary angiography.

## Introduction

Transradial access (TRA) has become the preferred vascular approach for diagnostic coronary angiography and percutaneous coronary intervention given its well-established advantages over transfemoral access. Numerous randomized clinical trials and international guideline recommendations have demonstrated that TRA significantly reduces major bleeding, vascular complications, and all-cause mortality in patients presenting with acute coronary syndromes, while also improving patient comfort, facilitating earlier mobilization, and shortening hospital stay. Consequently, interventional cardiology guidelines recommend a radial-first strategy whenever feasible [1,2].

Despite these advantages, successful transradial procedures depend on suitable vascular anatomy. Failure of radial artery cannulation may occur because of arterial spasm, vessel tortuosity, hypoplastic arteries, previous radial artery occlusion, severe calcification, or congenital anatomical variations. Such situations often necessitate crossover to an alternative vascular access site, most commonly the femoral artery [2,3].

The true superficial radial artery (SRA) is an uncommon congenital anatomical variant. Its reported prevalence is approximately 1%-1.5% [3]. During embryological development, the upper-limb arterial system develops from a primitive vascular plexus that undergoes progressive remodeling. Persistence of a superficial arterial channel may result in a radial artery that follows a superficial course over the dorsolateral aspect of the distal forearm and wrist rather than its usual volar anatomical pathway [3]. This anatomical variant should not be confused with distal transradial access (dTRA) through the anatomical snuffbox, which represents a distal puncture site of the radial artery rather than an anomalous superficial course of the vessel.

Consequently, the radial pulse may be absent or weak at the conventional puncture site despite preservation of distal perfusion, while a palpable pulse may be identified over the dorsolateral wrist. Careful physical examination combined with vascular ultrasound allows accurate identification of this unusual vascular anatomy. Ultrasound can assess the vessel’s course, diameter, depth, and patency, helping to determine its suitability for TRA and facilitating ultrasound-guided puncture [4,5].

We report a case of successful ultrasound-guided diagnostic coronary angiography performed through a true SRA in a patient with complete absence of the conventional radial pulse. This case underscores the importance of meticulous vascular examination and the value of ultrasound-guided access in the presence of rare arterial anatomy, and reinforces the need to consider SRA access before abandoning the transradial approach in favor of femoral access.

## Case presentation

A 62-year-old man with a medical history of type 2 diabetes mellitus, hypertension, and active cigarette smoking was referred to our cardiology service for evaluation of exertional chest discomfort. He described a several-month history of central chest tightness occurring during moderate physical activity and consistently relieved by rest, consistent with Canadian Cardiovascular Society (CCS) class II stable angina [6]. There was no history of prolonged resting chest pain, syncope, palpitations, orthopnea, paroxysmal nocturnal dyspnea, or previous myocardial infarction. There was no previous history of coronary revascularization or vascular intervention.

On admission, the patient was hemodynamically stable. Blood pressure was 130/85 mmHg, heart rate was 58 beats/min, respiratory rate was 15 breaths/min, and oxygen saturation was 96% on room air. Cardiovascular examination revealed normal heart sounds without murmurs or signs of heart failure. Baseline electrocardiogram (ECG) demonstrated sinus rhythm at 58 beats/min, with no significant ST-segment deviation or dynamic ischemic changes (Figure 1).

**Figure 1:**
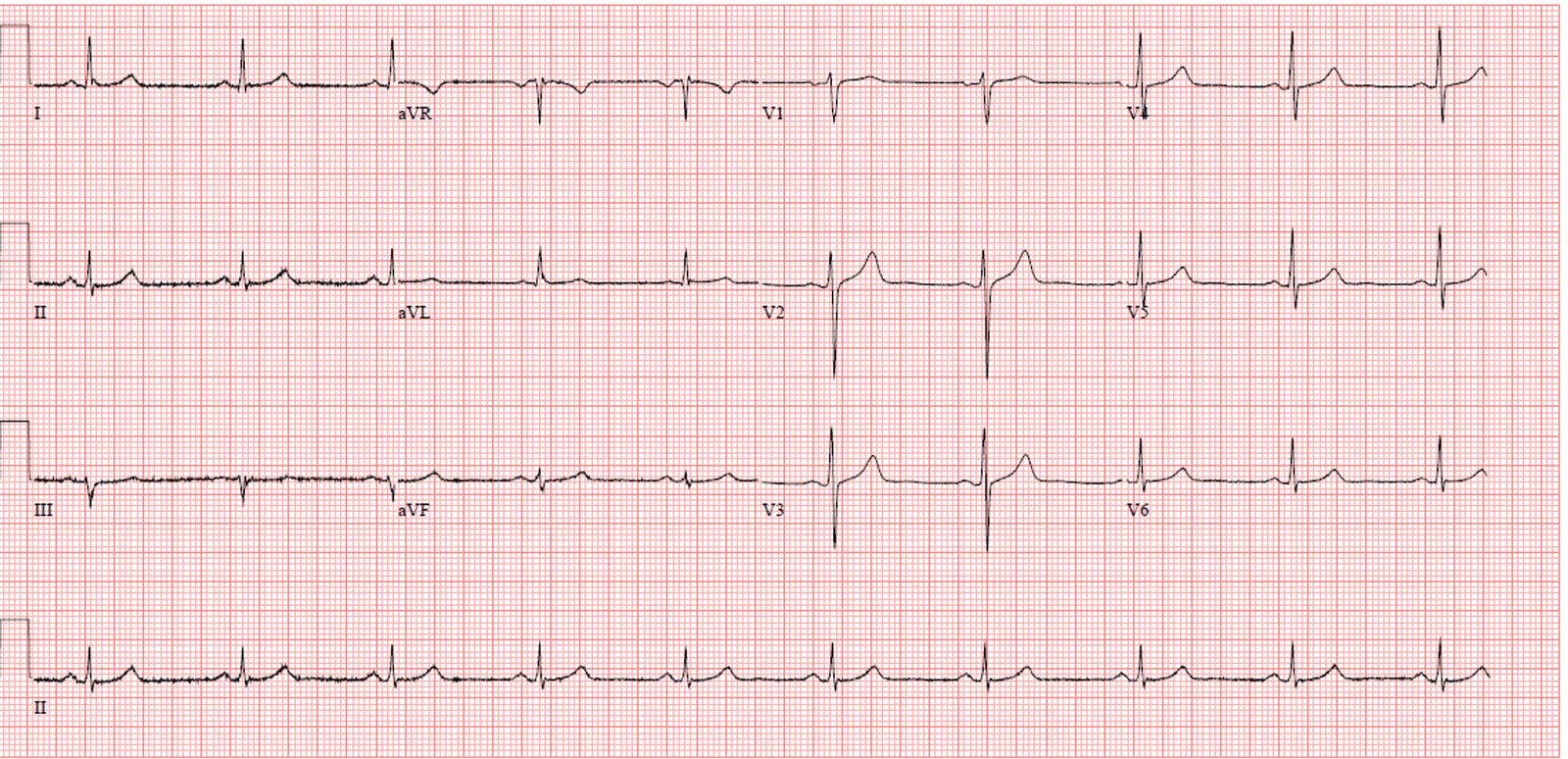
ECG: sinus rhythm, normal PR interval, and no ST-segment or T-wave changes. ECG, electrocardiogram

Transthoracic echocardiography demonstrated preserved left ventricular systolic function with an estimated left ventricular ejection fraction of approximately 55%. Mild hypokinesia of the inferior wall was noted without significant valvular heart disease or pericardial effusion (Figures 2-3; Video 1). In view of the patient's persistent exertional symptoms, cardiovascular risk profile, and echocardiographic findings, invasive coronary angiography was recommended.

**Figure 2:**
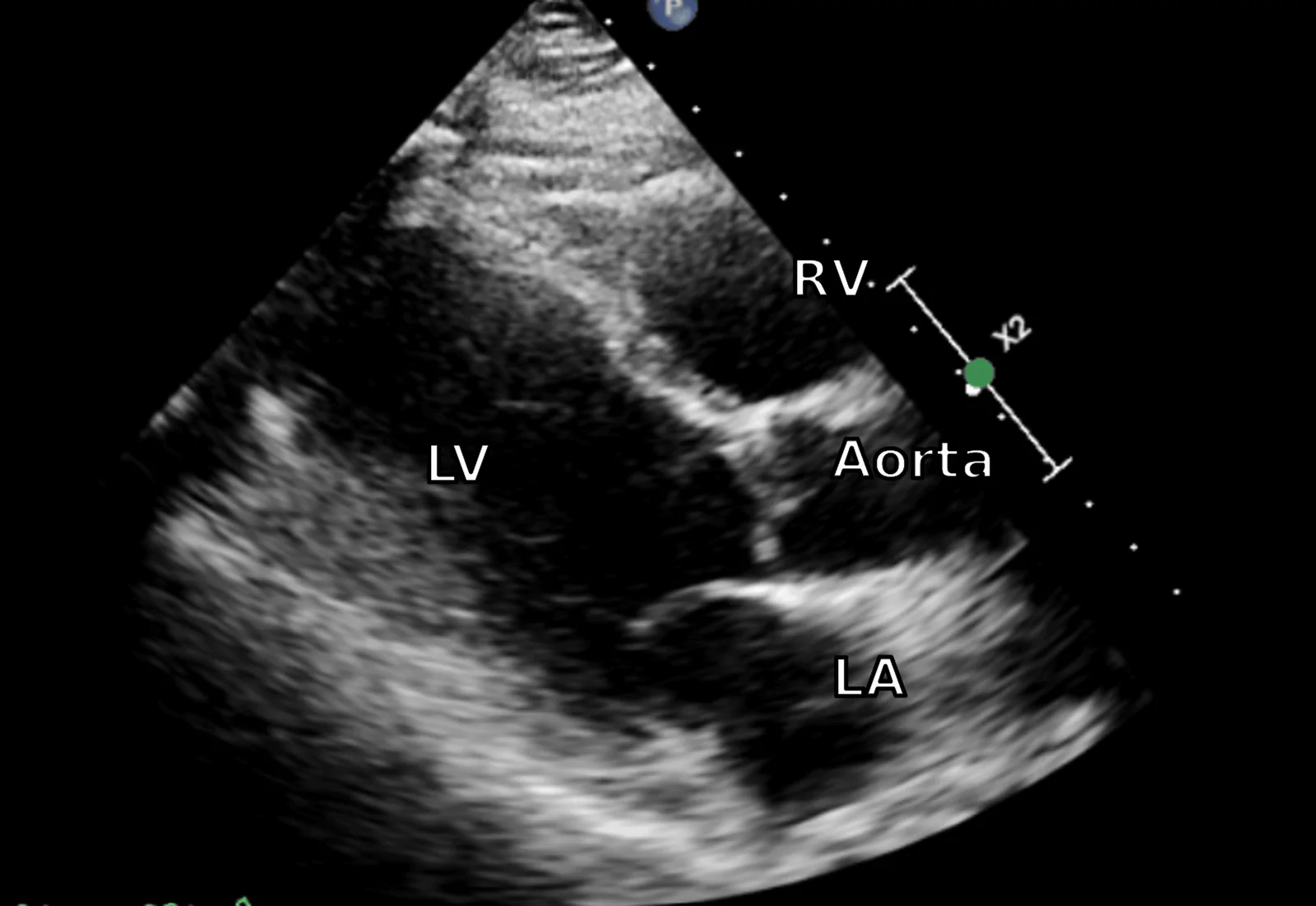
Two-dimensional echocardiography, parasternal long-axis view, showing LA, aorta, RV, and LV. LA, left atrium; LV, left ventricle; RV, right ventricle

**Figure 3:**
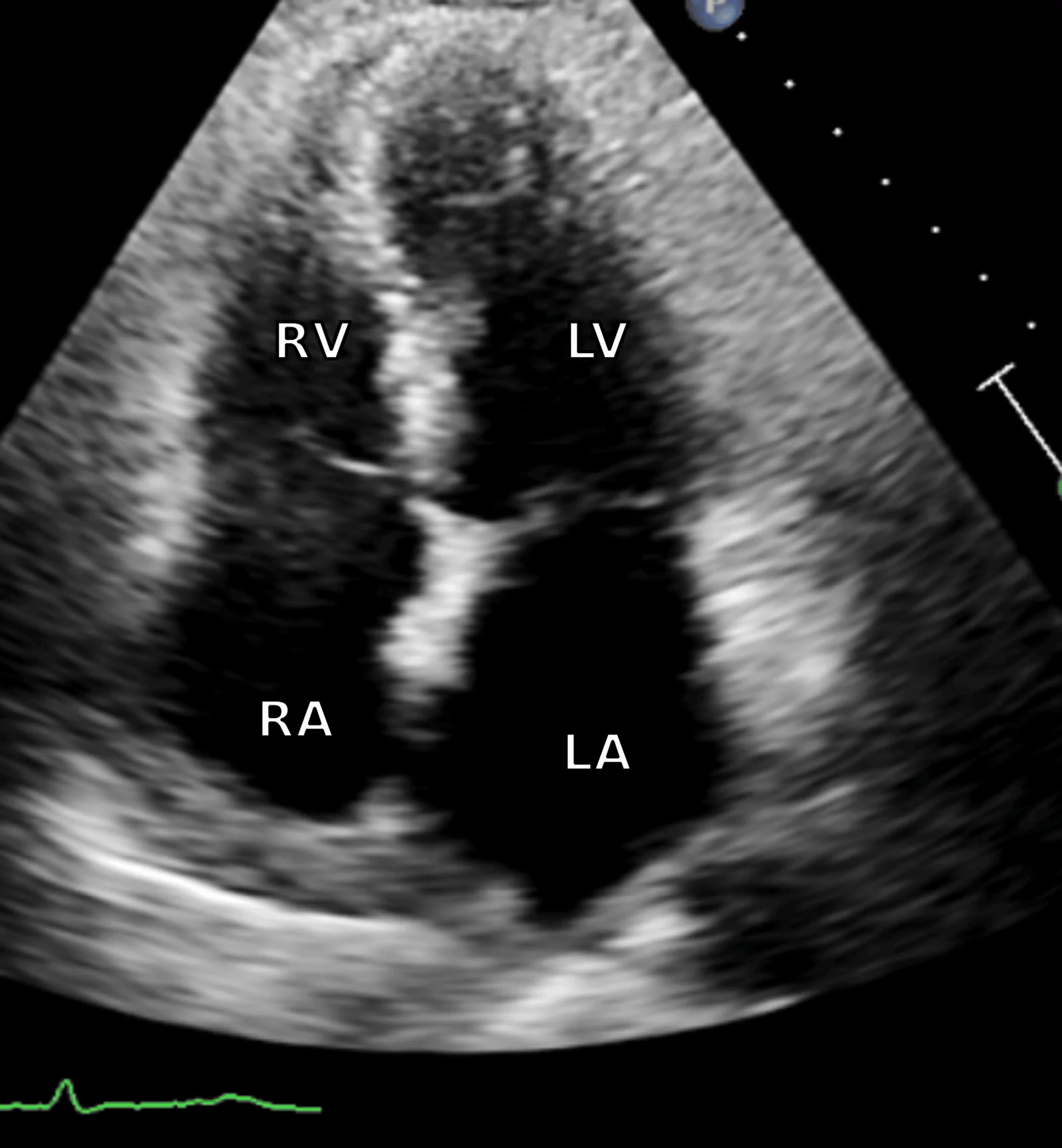
Two-dimensional transthoracic echocardiography, apical four-chamber view, showing a static frame of LA, RA, LV, and RV. LA, left atrium; LV, left ventricle; RA, right atrium; RV, right ventricle

**Video 1:** Two-dimensional transthoracic echocardiography, apical four-chamber view, showing normal left ventricular systolic function and normal right ventricular systolic function.

A focused vascular examination of both upper limbs was performed before coronary angiography. The right radial pulse was completely absent at the conventional volar wrist puncture site despite normal hand perfusion. The ulnar artery pulse was preserved. Careful palpation over the dorsolateral aspect of the wrist revealed a well-defined arterial pulsation following the anatomical course of an SRA. Examination of the left upper limb demonstrated a normal, palpable conventional radial pulse.

Bedside vascular ultrasound of the right wrist confirmed the presence of a true SRA traveling superficially over the dorsolateral wrist rather than following the usual deep anatomical course. The vessel demonstrated an adequate internal diameter for vascular access with normal arterial flow on color Doppler imaging. No evidence of radial artery occlusion, thrombosis, calcification, aneurysm, or significant arterial disease was identified. The conventional radial artery was absent at the usual puncture site, confirming the anatomical variant.

Following written informed consent, the procedure was performed under strict sterile conditions. Ultrasound-guided arterial puncture of the right SRA was performed using a 21-gauge arterial access needle. Access was obtained successfully on the first attempt without difficulty. A 6-French hydrophilic radial sheath was inserted smoothly over a 0.021-inch guidewire (Figure 4).

**Figure 4:**
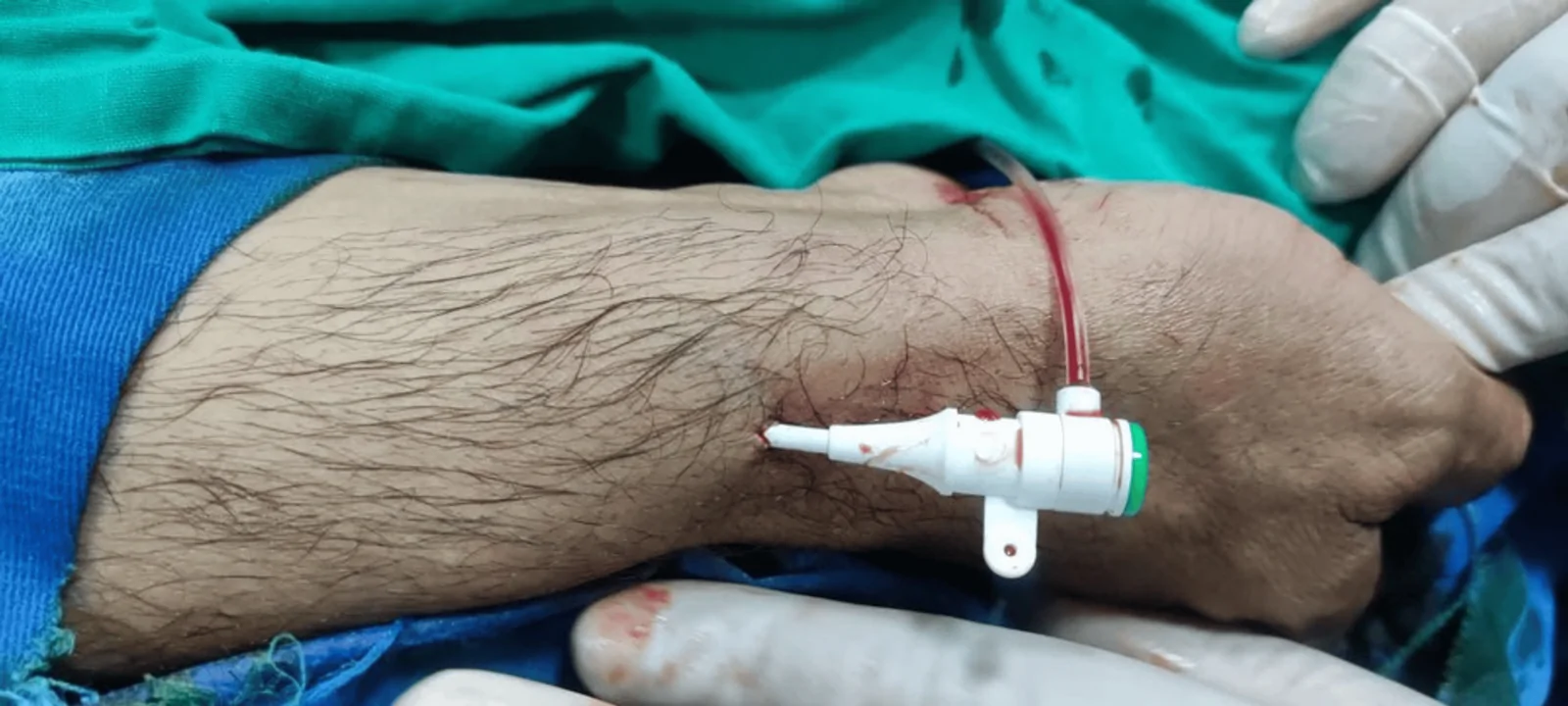
Radial sheath inserted in the superficial radial artery.

Immediately following sheath insertion, a transradial vasodilator cocktail comprising 100 µg of intra-arterial nitroglycerin and 2.5 mg of intra-arterial verapamil was administered. Intravenous unfractionated heparin (5,000 IU) was given according to institutional protocol.

Diagnostic coronary angiography was performed using a JR 3.5 diagnostic catheter. Catheter manipulation through the SRA, brachial artery, axillary artery, subclavian artery, and ascending aorta was straightforward, without excessive tortuosity or technical difficulty. No arterial spasm, resistance during catheter advancement, vascular injury, or access-site pain occurred during the procedure.

The total procedure duration was 15 minutes, fluoroscopy time was 10 minutes, contrast volume was 45 mL, and radiation exposure was 30 Gy·cm² (dose area product).

Selective coronary angiography demonstrated a right-dominant coronary circulation. The left main coronary artery was free from significant disease. The left anterior descending artery showed no significant stenosis. The left circumflex artery demonstrated a chronic total occlusion involving the proximal segment, extending into the second obtuse marginal branch (Figure 5; Video 2). Distal vessel opacification occurred through well-developed epicardial collateral channels arising from the right coronary artery, together with bridging collaterals across the occluded segment and good distal vessel runoff. The right coronary artery demonstrated no significant obstructive disease (Figure 6).

**Figure 5:**
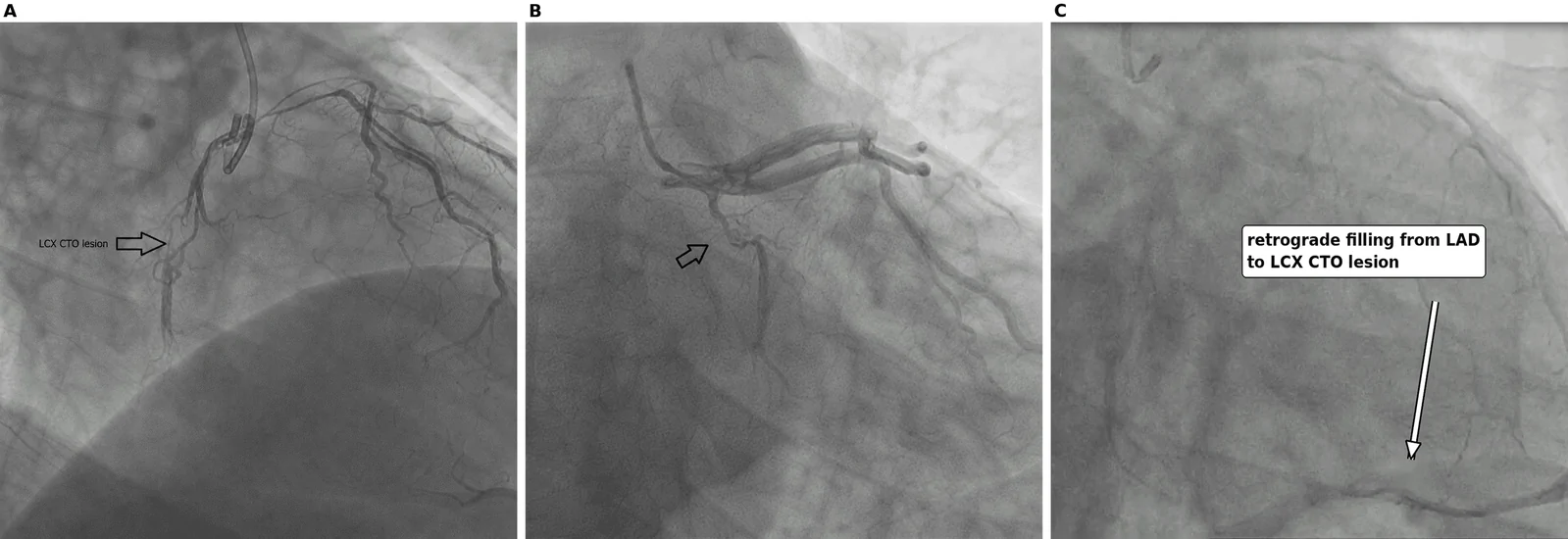
CTO of the LCX with retrograde collateral filling. (A, B) Coronary angiographic views demonstrating CTO of the LCX (arrows). (C) Coronary angiography demonstrating retrograde filling of the LCX from the LAD through collateral channels (arrow). CTO, chronic total occlusion; LAD, left anterior descending artery; LCX, left circumflex artery

**Video 2:** Coronary angiography demonstrating chronic total occlusion of the left circumflex artery. Coronary angiography demonstrating chronic total occlusion (CTO) of the left circumflex artery (LCX), with retrograde filling of the LCX from the left anterior descending artery (LAD) and right coronary artery (RCA) through collateral channels.

**Figure 6:**
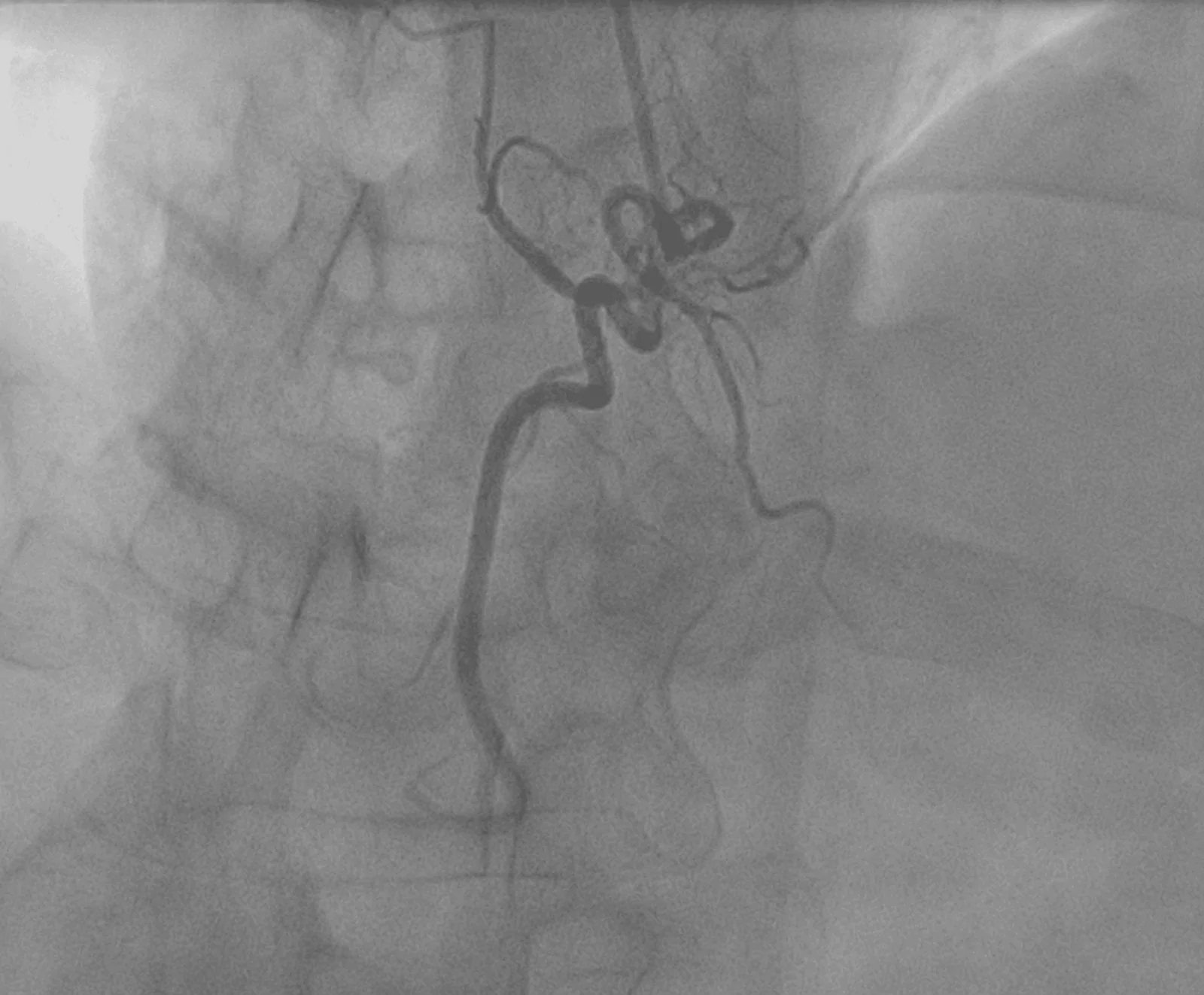
LAO view of the RCA showing no significant lesion. LAO, left anterior oblique view; RCA, right coronary artery

Given persistent symptoms despite optimal medical therapy and the anticipated technical complexity of CTO PCI, the case was discussed at the multidisciplinary revascularization meeting. In the absence of local dedicated CTO PCI expertise, the available treatment options were discussed with the patient, and a shared decision was made to refer him for consideration of coronary artery bypass grafting (CABG).

Following completion of the procedure, the radial sheath was removed, and hemostasis was achieved using manual compression, maintained for approximately 60 minutes according to the local transradial hemostasis protocol.

The patient remained hemodynamically stable throughout the procedure and recovery period. There were no immediate access-site complications, including radial artery spasm, bleeding, hematoma, pseudoaneurysm, arterial occlusion, hand ischemia, or neurological deficit. Distal perfusion of the hand remained intact with preservation of digital capillary refill.

At clinical follow-up two weeks later, the patient remained free from access-site complications. Examination demonstrated complete healing of the puncture site with no evidence of pain, infection, radial artery occlusion, or impairment of hand function. The patient subsequently proceeded with further surgical assessment for CABG.

## Discussion

TRA has become the preferred vascular approach for diagnostic coronary angiography and percutaneous coronary intervention owing to its established advantages over transfemoral access. These include lower rates of bleeding and vascular complications, earlier ambulation, greater patient comfort, and improved outcomes in patients with acute coronary syndromes. These benefits have established a radial-first strategy as the standard approach in contemporary interventional cardiology [1,2].

Our case illustrates the clinical importance of recognizing a true SRA. Complete absence of a palpable pulse at the conventional radial access site could easily have been interpreted as radial artery occlusion, severe arterial hypoplasia, or diffuse peripheral vascular disease, potentially prompting immediate conversion to femoral access. Instead, careful physical examination identified a distinct arterial pulse over the dorsolateral aspect of the wrist, and ultrasound examination confirmed the presence of a true SRA and enabled successful ultrasound-guided cannulation on the first attempt [3,4].

In patients undergoing coronary angiography, preprocedural radial artery ultrasound can also provide useful information regarding vessel suitability and the risk of vascular access complications [5].

Published experience with coronary catheterization through a true SRA remains extremely limited. Fu et al. described what they considered the first reported case of coronary angiography and percutaneous coronary intervention performed through an SRA in a 57-year-old man presenting with unstable angina [7].

In their case, the conventional radial pulse was weak but palpable, whereas the pulse over the anatomical snuffbox was significantly stronger. Color Doppler ultrasound confirmed the anatomical variation and guided successful distal radial puncture using a 6-French sheath. Coronary angiography and intravascular ultrasound-guided percutaneous coronary intervention were completed without reported complications, and the patient was discharged the following day [7].

Although our case shares several similarities with the report by Fu et al., including successful ultrasound-guided cannulation and uncomplicated completion of coronary catheterization, it highlights a different clinical presentation [7]. Unlike the previously reported patient, our patient had complete absence of a palpable radial pulse at the conventional volar wrist site. This made recognition of the anatomical variation dependent on careful physical examination of the dorsolateral wrist before ultrasound confirmation.

This distinction is clinically important because complete absence of the conventional radial pulse is more likely to prompt operators to give up the radial approach and switch to femoral access. Our case therefore reinforces the importance of systematic examination of the dorsolateral wrist whenever the conventional radial pulse cannot be identified.

Another noteworthy observation is that the SRA accommodated a standard 6-French hydrophilic sheath without difficulty. Catheter advancement through the SRA into the brachial artery was smooth, and the procedure was completed without arterial spasm, bleeding, hematoma, vascular injury, or neurological complications. These findings support the concept that, following careful ultrasound assessment, a true SRA may represent a safe and effective vascular access route in selected patients.

Although coronary angiography in our patient demonstrated chronic total occlusion of the left circumflex artery with extensive collateral circulation from the right coronary artery, the principal educational value of this report lies in the recognition of an uncommon vascular anomaly rather than the angiographic findings themselves. Increased awareness of this rare anatomical variation, together with the use of ultrasound when radial anatomy is uncertain, may reduce unnecessary femoral crossover and preserve the established advantages of the transradial approach.

## Conclusions

This case demonstrates that TRA through a true SRA variant may be feasible and successful for diagnostic coronary angiography when the conventional radial pulse is absent. Absence of the conventional radial pulse does not necessarily preclude TRA, and careful examination of the dorsolateral wrist may identify this anatomical variant. Vascular ultrasound can confirm the arterial anatomy, assess vessel suitability, and guide puncture. Early recognition of an SRA may prevent access failure and unnecessary femoral crossover.
